# Temperature and discharge variations in natural mineral water springs due to climate variability: a case study in the Piedmont Alps (NW Italy)

**DOI:** 10.1007/s10653-021-00864-8

**Published:** 2021-03-03

**Authors:** Leone Bastiancich, Manuela Lasagna, Susanna Mancini, Mauro Falco, Domenico Antonio De Luca

**Affiliations:** 1 Earth Sciences Department, Turin University, via Valperga Caluso 35, Turin, Italy; 2 Direzione Ambiente, Energia e Territorio, Settore Tutela delle acque, Regione Piemonte, via Principe Amedeo 17, Turin, Italy

**Keywords:** Natural mineral water spring, Mountain aquifer resilience, Climate variability, Temperature, Discharge, Trend

## Abstract

In the context of global climate change, understanding the relationships between climate and groundwater is increasingly important. This study in the NW Alps represents the first regional-scale investigation of the groundwater feature variation in mountain aquifers due to climate variability. The analysis of groundwater temperature and discharge in 28 natural mineral water springs and meteorological parameters (rainfall and air temperature) permitted us to evaluate the annual behaviour and possible trends of these parameters during the period from 2001 to 2018. The air temperature showed a positive trend almost everywhere, with a rise of up to 0.03 °C/year. In contrast, only ten springs showed a positive trend for groundwater temperature, but with the smallest rates of increase. Moreover, despite the substantial stability of the rainfall amount, 50% of the analysed springs showed a trend (29 and 21% for positive and negative trends, respectively) with low discharge variations. Finally, cross-correlation analyses proved the close relationship between air and groundwater temperatures, with a time lag between 0 and 3 months, and between spring discharge and air temperature, with a time lag between 1 and 3 months. In particular, spring discharge is closely connected to snow melting in spring and subordinate to rainfall. These results highlight the existing correlations between spring discharge and various meteorological and topographic parameters in the studied mountain area and provide a preliminary framework of the impacts of climatic variability on the availability and temperature of the exploited water resources.

**Supplementary Information:**

The online version contains supplementary material available at 10.1007/s10653-021-00864-8.

## Introduction

Climate change includes not only an increase in global temperatures but also changes in precipitation regime and the intensification of extreme events (i.e. flood and drought phenomena). It also has impacts on ecosystems, humans, animals, plants, and surface and groundwater environments. According to the Intergovernmental Panel on Climate Change (IPCC), the mean temperature is expected to increase globally throughout the twenty-first century, and the World Meteorological Organization (WMO) reported that the five-year period 2015–2019 is likely to be the warmest five-year period on record globally (IPCC 2018; WMO, 2019).

The Mediterranean region is currently experiencing this climate change. Average temperatures have already risen by 1.4 °C since the pre-industrial era in this area, indicating a warming 0.4 °C higher than the global average. It has consequences on various levels, such as rising sea levels (6 mm in 20 years) and increasingly extreme meteorological phenomena and drought (Cramer et al. 2018). Moreover, a pronounced decrease in precipitation, especially in the warm season, was observed in the southern Mediterranean areas and in the southern Alps (Brunetti et al. 2006a; Giorgi et al. 2008). Brunetti et al. (2006b) highlighted a strong positive trend of maximum temperatures in the last 50 years and a low and rare significant negative trend of precipitation (5% per century on a yearly basis). A general increase in trends for short-duration heavy rainfall in the Tuscany, Sicily, Lombardy and Piedmont regions has also been found (Arnone et al. 2013; Crisci et al. 2002; Saidi et al. 2015; Uboldi & Lussana, 2018).

Not only plains areas but also mountainous areas suffer from this situation. A high positive trend in air temperatures in alpine regions was observed, which reached and exceeded 2 °C in some cases (Jungo & Beniston, 2001). For snowfall in northern Italy, several studies show that the increase in temperatures has led to a drastic reduction in snow cover in alpine areas as well as on the surfaces of glaciers and permafrost. These degradations have led to a halving of the surfaces covered by glaciers in the last half century, from 4500 km^2^ recorded in 1850 to 2270 km^2^ recorded in 2000 (Mercalli et al. 2008). Terzago et al. (2013) showed how snowfall, over the period of 1951–2010, experienced a slight decrease of −0.2 cm/year at low-altitude stations and up to −1.4 cm/year at high altitudes. It was also observed that the spring season in particular contributed to this negative trend. Valt and Cianfarra (2014) detected decreasing trends in snow cover duration and snowfall during the last 40 years, and they found a strong linear correlation between snow duration and temperature during the springtime (March–April).

Regarding the effects of climate change on groundwater, many studies have shown that climate change has important repercussions, in terms of both quality and quantity (Doll and Florke 2005; Bloomfield et al. 2006; Green et al. 2011; Stuart et al. 2011; Taylor et al., 2012; Lasagna, Ducci, et al., 2020; Grappein et al. 2021). More specifically, groundwater recharge depends on the distribution, amount and timing of precipitation; evapotranspiration losses; losses from watercourses; snow cover thickness; snow melt characteristics; land cover and air temperature (Taylor et al. 2012). Doll and Florke (2005) modelled recharge by applying four climate change scenarios, and they observed a large decrease (up to 70%) in some currently semi-arid zones (including the Mediterranean area) and an increase (locally higher than 30%) in other large regions, including the Sahel, northern China, the western United States and Siberia.

Many studies performed in different parts of the world, such as the Indian subcontinent (Rodell et al. 2009; Tiwari et al. 2009), North Africa (Lutz et al. 2016) and different parts of the USA (Zwilling et al. 1989), show general groundwater depletion due to the overlapping effects of climatic variability and anthropogenic factors (such as overpumping).

In southern Italy, a decreasing trend in annual rainfall, notable after 1980, has been observed, and consequently, a widespread dramatic decrease in groundwater availability has been observed due to the overlapping effects of water demand and climate change (Ducci & Polemio, 2018; Polemio & Casarano, 2008).

In the Piedmont Plain (north-western Italy), Lasagna et al. (2019), Lasagna, Mancini, et al. (2020)) observed, in almost all the alluvial plains, the dependence of the water table level on climate variability, with a higher piezometric level due to the highest rainfall occurrence in the 2009–2017 period.

Regarding the groundwater temperature, an increase in water resource temperatures has been observed due to rapid climate warming (Bloomfield et al. 2013; Gunawardhana & Kazama, 2011).

However, while the effects of climate change or variability on plain aquifers have been commonly observed and recognized in the scientific literature, there is still a lack of knowledge on the effects on mountain aquifers and springs. The variation in meteorological parameters has important consequences for the hydrological cycle in mountainous regions, where the water supply is dominated by melting snow or ice (Baernett et al. 2005; Bavay et al. 2009). As highlighted in some studies (Houben et al. 2014; Menberg et al. 2014), groundwater in plain aquifers responds to climate change with relatively long response times. In contrast, spring waters in particular contexts (e.g. karst springs) respond faster to climate change (Vigna & Banzato, 2015). Recent studies in southern Italy highlighted a decrease in some karst spring discharge since 1987, with reductions ranging from 15 to 30%, followed by an attenuation of the negative trend and, in some cases, a reversal of the trend (Fiorillo et al. 2015). Mastrocicco et al. (2019) showed that the Campania region (South Italy) experienced an increase in spring water temperatures of approximately 2.0 °C during the monitored period (2002–2017). The rainfall–runoff model and statistical analysis applied to discharge data of Italian Northern Apennines springs (Cervi et al. 2018) show no evidence of change in mean annual discharge. However, the estimated reduction in effective rainfall from June to November will likely lead to a significant decrease in future discharges over the same period (up to 26.3%). Moreover, the low-flow lengths are also affected, with more years in the future presenting exceptionally long continuous low flows, indicating a significant impact of climate change on the spring regime.

In this paper, 28 natural mineral water springs located in the alpine mountains in NW Italy are analysed to provide a framework of the possible impact of climatic variability (according the definition of the IPCC, 2014) on the availability and features of exploited water resources. More specifically, time series of springs and meteorological parameters from 2001 to 2018 are studied to evaluate annual behaviour. Moreover, trend and cross-correlation analyses are used to evaluate whether climatic variability has had any consequences on spring water temperature and discharge. This study represents the first regional-scale investigation in the NW Alps of the groundwater feature variation in mountain aquifers due to climate variability. Moreover, as natural mineral water has wide public and large economic interests related to the marketing of bottled water (Ciotoli & Guerra, 2016), the investigation of possible trends in quality and quantity could provide useful information for the owners and stakeholders of bottled mineral water brands as well as for the whole community.

## Study area

**Fig. 1:**
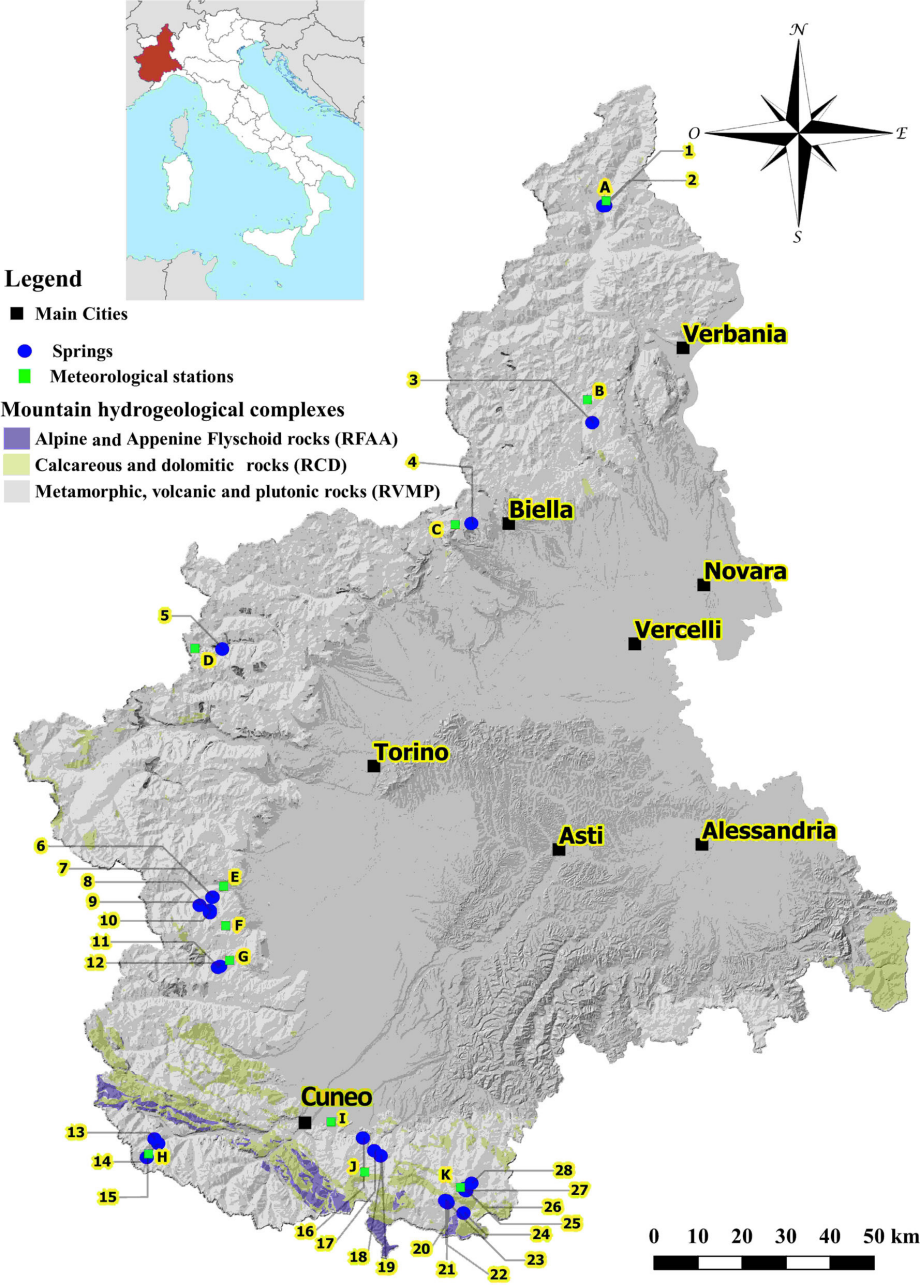
Location of the analysed springs in the Piedmont Region mountainous area and associated hydrogeological complexes. The quaternary superficial deposit complex is not reported in the map due to its large distribution. The grey colour indicates the plain and hilly areas not analysed in the paper; the numbers and letters represent the denomination of the studied springs and meteorological stations, respectively

In this study, 28 springs located in the mountainous area of the Piedmont region are analysed (NW Italy). The Piedmont is located at the head of the Po Valley, and it is limited on three sides by mountains (the Alpine chain). It extends for approximately 25,400 square kilometres and consists of mountains for approximately 43% of its territory, hills for 30% and plains for the remaining 27%. The analysed springs are located between altitudes of 465 m above sea level (a.s.l.) and 2150 m a.s.l. in the mountainous area (Fig. 1).

The mountainous areas of the Piedmont region are characterized by four hydrogeological complexes: calcareous and dolomitic rocks (RCD), alpine and apennine flyschoid rocks (RFAA), metamorphic, volcanic and plutonic rocks (RVMP) and Quaternary superficial deposits (QSD) (De Luca et al. 2020; Piana et al. 2017).

Calcareous and dolomitic rocks (RCD) (Triassic–Paleogene) consist of limestone-dolomitic rocks and strongly tectonized evaporitic–carbonatic rocks (carbonate breccias) of the Alpine and Apennine substrata. This complex, which outcrops in the southern Piedmont, is characterized by marked water circulation due to the development of superficial and deep karst phenomena. In the most calcareous rocks, the prevalent permeability, due to fractures and karst, is high to medium; in the dolomitic rocks, the permeability and karst phenomena are smaller. The rocks often host springs with high discharge, frequently higher than 100 L/s (average annual flow). The groundwater quality is generally good, except in the presence of evaporitic rocks.

Alpine and Apennine Flyschoid rocks (RFAA) (Late Cretaceous–early Eocene) are alternations of argillaceous schists with clays, sandstones, limestones, argilloschists, slate schists and subordinate limestones and conglomerates (Alpine flyschoid rocks). Moreover, they are also represented by alternations of carbonates, calcareous marls, arenaceous layers, blackish clayey banks and grey-blackish argillites (Apennine flyschoid rocks). In both cases, the prevalent permeability is low or very low. The groundwater circulation, which is generally limited and of local importance, is related to the lithology and the degree of fracturing. This complex only outcrops in the southern Piedmont.

Metamorphic, volcanic and plutonic rocks (RVMP) (Paleozoic–Cenozoic) are the magmatic (plutonic and volcanic) and metamorphic rocks of the Alpine and Apennine substrata. They are gneisses, mica schists, quartzites, green stones (serpentinites, amphibolites and prasinites), granites, porphyries and their metamorphic derivatives. This complex is predominant in the Piedmont region. Groundwater circulation is absent or limited to surface fracture systems and significant faults. The prevalent permeability varies from low to very low. The degree of permeability can be medium with the most fractured bands. Springs are characterized by modest flow rates (a few L/s) and good quality (natural mineral waters).

The quaternary superficial deposit complex (QSD) consists of continental deposits (colluvial, gravitational and periglacial) located in the mountainous and hilly sectors of the Piedmont. They can be generically considered “ubiquitous deposits” sensu Polino et al. (2002) and Bini et al. (2004). These include glacial deposits preserved along valley sides and terraced alluvial sediments located along riverbeds. The prevalent permeability due to porosity varies from medium to low, with local highs.

From a geological point of view, the analysed springs are located in areas characterized by a fractured crystalline substrate covered by quaternary superficial deposits. Circulation generally occurs in the cover and subordinately in the upper part of the fractured substrate (Clemente et al. 2008; De Luca et al. 2015, 2019).

The average annual precipitation in the Piedmont region over the 1957–2009 period shows values higher than 1600 mm/year in the northern mountainous part of the region and quantities less than 720 mm/year in the south-eastern plain sector (http://rsaonline.arpa.piemonte.it/meteoclima50/clima_ed_indicatori.htm). The annual rainfall distribution is characterized by two maxima (spring and autumn) and two minima (winter and summer) (Acquaotta & Fratianni, 2013; Biancotti et al. 1998). The analysis of the annual precipitation in the Piedmont region over the period of 2001–2015 shows some modifications compared to the previous period of 1971–2000. More specifically, an increase was observed in the Verbano area, in correspondence with the area of Lake Maggiore, and a slight overall decrease was detected in the rest of the region, which was more significant in the Biella area and in the southern zone between Cuneo and Alessandria (https://www.arpa.piemonte.it/rischinaturali/tematismi/clima/confronti-storici/analisi-lungo.html). Moreover, an increase in intense events was detected where the annual rainfall increased.

The average annual temperature over the period of 1958–2009 highlighted the following minimum and maximum values, categorized by altimetric ranges: 8.2–16.9 °C at altitudes less than 500 m; 5.2–12.6 °C in the range of 500–1500 m; 0.8–6.5 °C in the range of 1500–2500 m; and −3.9–0.3 °C at altitudes higher than 2500 m (http://rsaonline.arpa.piemonte.it/meteoclima50/clima_ed_indicatori.htm). The hottest month of the year is July, while the coldest month is January. The analysis of the annual average temperature in the Piedmont region over the period of 1958—2015 shows a positive trend. In particular, in recent years, the annual average temperature has always been above that in the reference period (1971–2000), with an estimated total increase of approximately 1 °C in 50 years. An important trend was also identified in the maximum temperature values, with an increase of 2 °C in approximately 60 years. (http://rsaonline.arpa.piemonte.it/meteoclima50/clima_ed_indicatori.htm).

A recent study in the Piedmont Plain (Lasagna, Mancini, et al., 2020) analysed rainfall time series over the period of 2002–2017 and highlighted the presence of a change point in 2008, detected in 88% of the rainfall time series, after which an increase in precipitation was observed.

The studied snow in the mountainous areas showed negative anomalies at all the analysed stations, with a few isolated exceptions, for the snow height and snowy days from 1980 to 2010 (ARPA, 2013).

### The Piedmont mineral water springs

**Fig. 2:**
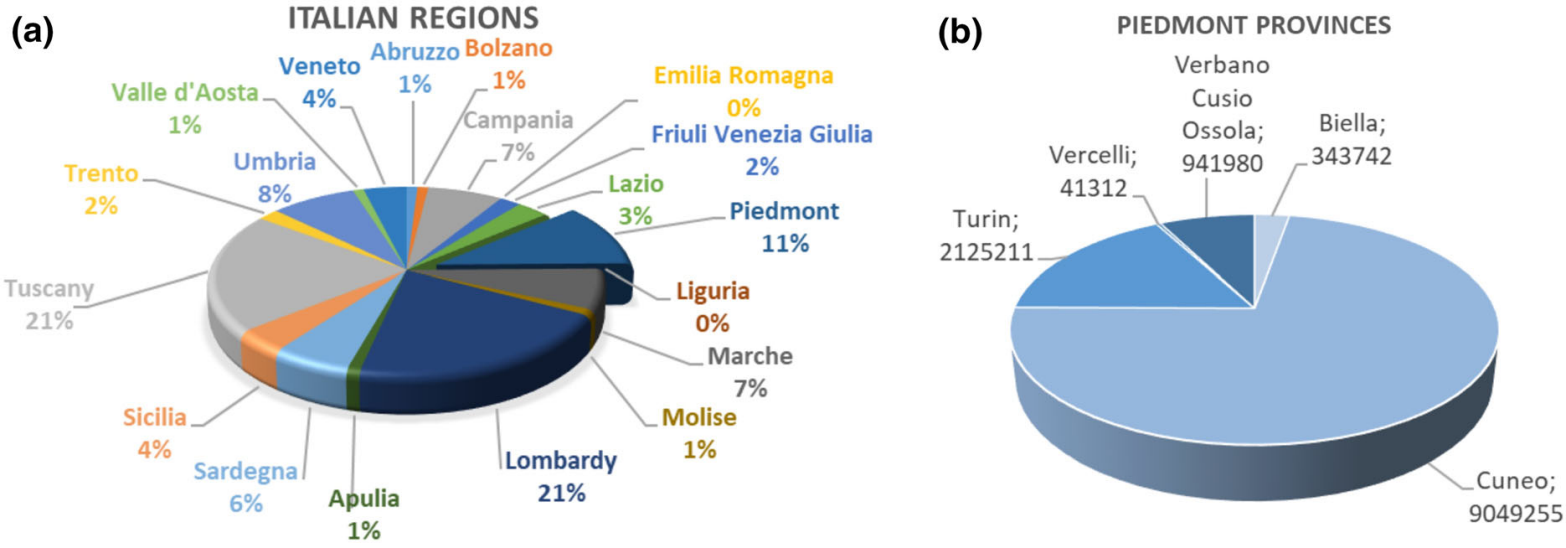
Percentage of mineral water sources (number of springs plus wells) divided by Italian region (**a**), and annual volume (m^3^/year) supplied by the springs in the provinces of the Piedmont (**b**)

In this study, natural mineral water springs used for bottled water in the Piedmont Region are analysed. The consumption of bottled natural mineral water has grown enormously in Italy, and Northwest Italy is currently the leader in its distribution and consumption (30%) (Beverfood, 2012; Ciotoli & Guerra, 2016). At a national level, the Piedmont region has the highest number of mineral springs, followed by Lombardy, Tuscany and Veneto. Moreover, the Piedmont is the third region in terms of the mineral water sources (number of springs plus wells) and for the presence of brands for bottled mineral water (Fig. 2a). In detail, Cuneo Province provides the largest quantity of bottled mineral water in the Piedmont region (Fig. 2b). Finally, in the Piedmont, exploitation of mineral water is prevalent respect to thermal water (Table 1). These data highlight the high value, both in economic and environmental terms, of Piedmont natural mineral water springs in the Italian panorama.

**Table 1:** Distribution of concessions and research permits of mineral water and thermal water in the Piedmont

	Mineral waters		Thermo-mineral waters	
Province	Concessions	Research permits	Concessions	Research permits
Alessandria	6	0	4	0
Asti	0	0	1	1
Biella	3	3	0	0
Cuneo	18	11	2	1
Novara	0	0	0	0
Turin	9	1	1	2
Verbania	7	0	3	2
Vercelli	1	0	0	0
Total	44	15	11	6

**Fig. 3:**
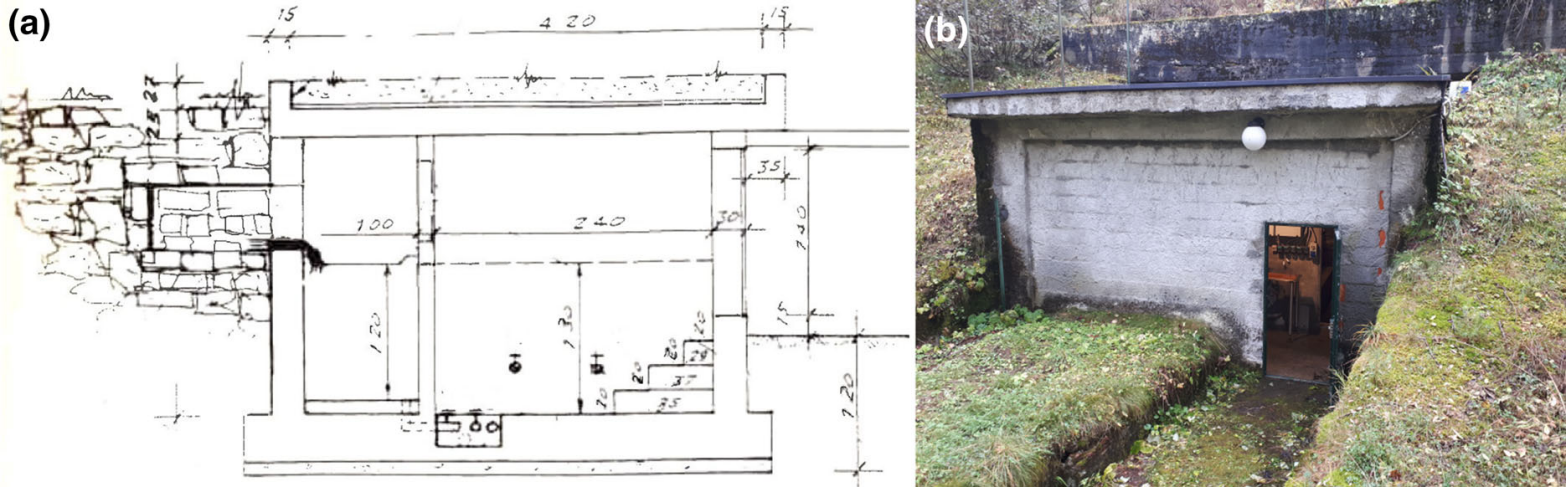
**a** Original scheme of the Spring 15 capture structure. The numbers refer to the size in cm. **b** Picture of the spring capture structure at Spring 5

All 28 studied springs are gravity springs, and groundwater naturally flows in a capture structure without pumping. The capture structure accumulates groundwater as it emerges at the head of the spring. A typical spring capture structure is a collection chamber consisting of a simple concrete box laterally tied directly to the rock formation yielding groundwater. The wall coupled with the aquifer is perforated to allow inflow. The water enters the first chamber, called the sediment box, where fine particles settle out of the water. A weir and a temperature data logger are generally located in the sediment box. Then, groundwater overflows through the weir into a clear water reservoir, where pipes deliver it into the distribution network. Protective drainage ditches are generally present to keep superficial drainage water a safe distance from the spring. An example of a spring capture structure is shown in Fig. 3.

## Materials and methods

### Database and elaborations

Discharge (Qgw) and water temperature (Tgw) data of the springs were elaborated. Data are from the regional monitoring network of mineral waters collected in a dedicated database (Piedmont region mineral water monitoring database). The monitored periods vary between 8 and 18 years, depending on the spring (Table 1). The database complies with the current regional legislation (l.r. 25/1994) that establishes the regulatory requirements for the mineral water concessionaires to collect and communicate, every six months, the following data, for both springs and wells: i) daily flow rate, temperature and electrical conductivity for each withdrawal point in the concession and ii) daily rainfall and air temperature. The spring data are collected by automatic instruments with continuous recording: a submersible level sensor, with an accuracy of ± 1 mm, for Qgw and a resistance temperature detector, with an accuracy of 0.1 °C, for Tgw. Qgw and Tgw in the analysed springs were measured six times per day. The daily data were calculated as the average measurement for a 24 h period. Then, Qgw and Tgw data were aggregated monthly and yearly for the subsequent analyses. The continuity (ICON) and completeness (ICOM) of the data series (Qgw and Tgw) were evaluated using the following equations:

1 $$ICON = 1 - 2 \times \frac{{\text{number of missing data ranges}}}{{\text{maximum number of data}}} \times 100$$

2 $$ICOM = \frac{{\text{number of valid data}}}{{\text{maximum number of data}}} \times 100$$

An analysis of the spring altitude and the surface and maximum altitudes of the water catchment area located at the highest elevation above the spring (WCS) was also performed. Moreover, spring data were evaluated to identify the minimum, average and maximum daily Qgw and Tgw in the analysed period.

Correlation analyses between the average annual Tgw and spring altitude and the average annual Tgw and maximum altitude of the WCS were carried out using biplots.

Finally, Qgw variability was analysed using the Meinzer variability index R (Meinzer, 1923), according to the equation:

3 $$R = \frac{{Q_{{{\text{max}}}} - Q_{{{\text{min}}}} }}{{Q_{m} }} \cdot 100$$

where Qmax, Qmin and Qm are the maximum, minimum and mean monthly discharge in the analysed period, respectively. Thus, a variable spring is one in which the index number is 100% or more. A subvariable spring is one in which the index number is between 25 and 100%, and a constant spring is one in which the index number is not more than 25% (Meinzer, 1923).

Rainfall and air temperature (Tair) were also analysed. These data are from the online database of Arpa Piemonte (Agenzia Regionale per la Protezione Ambientale—Environmental Protection Agency of Piedmont Region) (https://www.arpa.piemonte.it/rischinaturali/accesso-ai-dati/annali_meteoidrologici/annali-meteo-idro/banca-dati-meteorologica.html). More specifically, the meteorological parameter data were collected in correspondence with 11 weather stations. The daily data were aggregated monthly and annually. The minimum, maximum, and average monthly Tair in the whole analysed time period were computed.

The springs and meteorological parameters were also analysed by plotting daily Qgw, Tgw, and rainfall in a diagram for each spring to identify the annual behaviour of these parameters.

### Statistical analyses

Different statistical analyses were conducted to better understand the Qgw and Tgw trends of the analysed springs and the possible links with the climatic variables (Tair and rainfall).

#### Trend analysis

Trend analysis of hydrological variables (Qgw and Tgw of springs) and meteorological time series (rainfall and Tair) was conducted using monthly aggregated data (monthly cumulative rainfall, average monthly Tair, Tgw, and Qgw) for the period of 2001–2018. The Mann–Kendall test (MKT) was applied to verify the existence of a monotonic trend (Hirsch et al. 1982). The MKT is a nonparametric test, and no assumption of normality is required. It has been widely applied to assess the significance of monotonic trends in hydrological and climatological time series. Moreover, the MKT is useful because its statistic is based on (positive or negative) signs, and therefore, the trends determined are less affected by outliers (Birsan et al. 2005). In the MKT, the null hypothesis, H0, indicates that there is no monotonic trend in the series. The alternate hypothesis is that a trend exists. This trend can be positive, negative, or nonnull. In this study, the significance level of the statistical MKT was set at 0.05, and the calculated p value was compared to this threshold. To evaluate the sign of the trend, the tau index was used; consequently, in the presence of a positive tau, the trend will be positive, and vice versa in the case of a negative tau.

#### Cross-correlation analysis

In this study, cross-correlation analysis was applied to i) Tair and Tgw and ii) Qgw and Tair. Cross-correlation between rainfall and Qgw was not performed because, locally, we did not have total snowfall data.

Using the cross-correlation technique, two series are compared to identify the position over time of the observations in which there is a clear correspondence between the series. This comparison provides two parameters: the strength and the sign of the correlation between the two series and the lag (or time shift or translation), which corresponds to the position of maximum equivalence between the series. It uses value functions of the Pearson correlation coefficient (r) for two time series shifted in relation to each other by time unit Δt (Graf, 2019). Investigating cross-correlations between hydro-meteorological signals grants a deep understanding of the whole hydrological system and management of water resources (Ruigar & Golian, 2015). Many studies have applied cross-correlation analyses to meteorological, climatical and hydrological time series (Cheng et al. 2019; Sivakumar, 2005; Vassoler & Zebende, 2012). Fiorillo and Doglioni (2010) applied a cross-correlation analysis to rainfall and spring discharge to detect the time required for water to flow through karst aquifers in southern Italy. The cross-correlation function and its decay were used to assess the character, strength, and direction of the relationship between the water and air temperature time series (Graf, 2019).

Finally, the autocorrelation functions (ACFs) of Tair and Tgw were also analysed to assess whether the seasonal components found in the cross-correlations were present. The ACF allows the identification of the components that repeat systematically in specific time intervals (seasonality). The ACF expresses the linear link between each datum and those that precede it Table 2.

**Table 2:** Period of monitoring for the 28 studied springs and indication of the monitored years

Spring Code	Start of monitoring	End of monitoring	Monitored years
1	01/01/2001	01/12/2018	18
2	01/01/2001	01/12/2018	18
3	01/01/2002	01/12/2018	17
4	01/02/2002	01/12/2018	17
5	01/03/2001	01/12/2018	18
6	01/01/2008	01/12/2018	11
7	01/01/2008	01/12/2018	11
8	01/01/2008	01/12/2018	11
9	01/07/2005	01/12/2018	13
10	01/07/2005	01/12/2018	13
11	01/07/2010	01/12/2018	8
12	01/07/2010	01/12/2018	8
13	01/07/2002	01/12/2018	16
14	01/09/2004	01/12/2018	14
15	01/09/2004	01/12/2018	14
16	01/07/2004	01/12/2018	14
17	01/01/2005	01/12/2018	14
18	01/01/2001	01/12/2018	18
19	01/01/2001	01/12/2018	18
20	01/01/2002	01/12/2018	17
21	01/01/2002	01/12/2018	17
22	01/01/2002	01/12/2018	17
23	01/01/2002	01/12/2018	17
24	01/07/2002	01/12/2018	16
25	01/01/2002	01/12/2018	17
26	01/01/2002	01/12/2018	17
27	01/01/2002	01/12/2018	17
28	01/01/2002	01/12/2018	17

## Results

### Springs and meteorological data

The analysed springs are located at different altitudes in the alpine mountain area of the Piedmont region. Most of the springs (54%) are located at an altitude between 1000 m a.s.l. and 1500 m a.s.l., 29% of the springs are located at an altitude higher than 1500 m a.s.l., and only 17% are situated at an altitude less than 1000 m a.s.l. (Table 3). The maximum altitude of the WCS is 2850 m a.s.l., while the minimum altitude is 812 m a.s.l. The surface of the WCS is highly variable, ranging from 0.34 to 11.58 km^2^.

**Table 3:** Main features of the analysed springs and spring data series

Spring code	Altitude m a.s.l	WCS maximum altitude m a.s.l	WCS surface km^2^	ICON %	ICOM %
1	465	2850	11.58	94	96
2	551	2850	11.58	90	90
3	704	1000	1.78	96	97
4	946	1725	1.28	100	100
5	1460	2350	2.13	98	95
6	650	1000	2.67	100	97
7	650	1000	2.67	100	97
8	1010	2100	4.67	100	100
9	1300	1885	1.07	99	94
10	1100	2100	4.67	99	98
11	2150	2350	1.87	100	100
12	2030	2350	1.87	100	100
13	1500	2300	1.40	99	97
14	1370	2700	0.34	99	98
15	1560	2676	1.40	100	100
16	605	812	1.34	100	100
17	1076	1761	3.21	99	100
18	1405	1510	1.01	100	100
19	1318	1510	1.01	99	100
20	1480	2150	3.70	100	100
21	1530	2150	3.70	100	100
22	1540	2150	3.70	100	100
23	1450	2150	3.70	100	100
24	727	1739	3.60	100	100
25	1355	1780	1.93	100	100
26	1358	1780	1.93	100	100
27	1282	1780	1.93	100	100
28	1090	1880	2.34	99	97

All the springs show a high continuity of data series (ICON > 90%) and a high completeness of data series (ICOM > 90%).

In Table 4, the values of minimum, average and maximum daily Qgw and Tgw of the springs are reported. All the studied springs can be classified as "cold", according to the Mouren classification (Schoeller, 1962). The Tgw is at a minimum in spring and a maximum in autumn.

**Table 4:** Minimum, maximum and average daily Qgw and Tgw in the analysed period

Spring code	Minimum Qgw, L/s	Average Qgw, L/s	Maximum Qgw, L/s	Minimum Tgw, °C	Average Tgw, °C	Maximum Tgw, °C
1	0.10	0.74	1.95	8.31	10.59	12.58
2	28.08	35.59	41.94	9.79	9.98	10.15
3	0.72	1.84	5.12	4.97	10.69	17.13
4	2.36	5.63	11.72	4.14	8.76	9.16
5	0.71	4.20	10.15	3.79	4.09	4.29
6	0.67	8.76	36.76	5.61	8.99	12.21
7	1.87	10.03	36.59	6.00	9.16	13.00
8	4.65	9.91	21.81	3.62	7.94	8.16
9	0.62	30.52	71.52	5.09	5.92	6.44
10	0.44	1.61	3.67	7.01	7.36	7.71
11	12.97	20.58	30.07	3.68	4.60	5.47
12	9.36	13.55	29.22	3.02	3.51	4.44
13	9.85	16.68	26.17	2.66	6.23	11.28
14	3.57	9.39	12.11	4.55	6.60	8.04
15	8.82	12.32	17.05	4.22	7.27	9.52
16	15.54	20.76	23.38	7.08	10.33	10.43
17	0.07	0.48	0.69	4.34	5.62	6.51
18	0.16	0.58	2.35	1.52	6.80	11.63
19	0.91	1.78	3.97	2.59	6.92	7.63
20	0.69	3.49	9.31	6.71	6.92	7.13
21	2.85	13.44	41.54	5.23	6.53	6.86
22	3.03	9.47	36.51	6.01	6.40	7.08
23	1.57	6.74	49.68	6.63	7.03	7.20
24	1.69	6.56	13.15	6.24	8.40	10.01
25	3.26	8.66	22.51	6.31	6.58	6.73
26	0.20	1.48	13.14	4.76	6.12	7.63
27	1.23	6.97	15.91	4.19	7.31	11.32
28	1.15	4.11	14.66	6.99	7.67	8.45

The 11 analysed weather stations are located at altitudes ranging between 470 m a.s.l. and 1765 a.s.l. (Table 5). Data from the weather stations show a very high continuity (ICON > 99%) and a completeness (ICOM) equal to 100%.

**Table 5:** Main features of the weather stations and the average, minimum and maximum monthly Tair during the period of 2001–2018

Weather station code	Location	Altitude m a.s.l	ICON %	ICOM %	Minimum Tair °C	Average Tair °C	Maximum Tair °C
A	Crodo	560	100	100	−0.15	11.16	23.87
B	Varallo	470	100	100	−2.10	10.46	23.27
C	Andrate Pinalba	1580	100	100	−4.05	6.79	19.05
D	Ala di Stura	1006	99	100	−5.11	6.56	18.13
E	Luserna San Giovanni	475	99	100	−1.23	11.40	24.79
F	Barge	961	99	100	−1.59	10.26	23.57
G	Paesana	1265	99	100	−2.81	9.11	21.74
H	Vinadio-San Bernolfo	1695	99	100	−4.36	6.95	19.17
I	Boves	575	100	100	−2.08	11.20	24.61
J	Chiusa Pesio	935	100	100	−4.02	8.29	21.01
K	Monte Berlino	1765	100	100	−6.32	5.58	17.54

The registered average monthly Tair ranges between 5.58 °C, in correspondence with the weather stations located at the highest altitude (i.e. weather station K), and 11.40 °C at the lowest altitude (i.e. weather station E). At these weather stations, the highest maximum Tair (24.79 °C at E) and the lowest maximum Tair (17.54 °C at K) were also registered (Table 5).

The maximum Tair is in the summer (July and August), and the lowest Tair is in the winter (December-January).

Regarding rainfall, the analysed weather stations show a bimodal trend, with two maxima and two minima. The maxima are in the spring (April–May) and autumn (November), while the minima are in the summer (July) and winter (January).

**Fig. 4:**
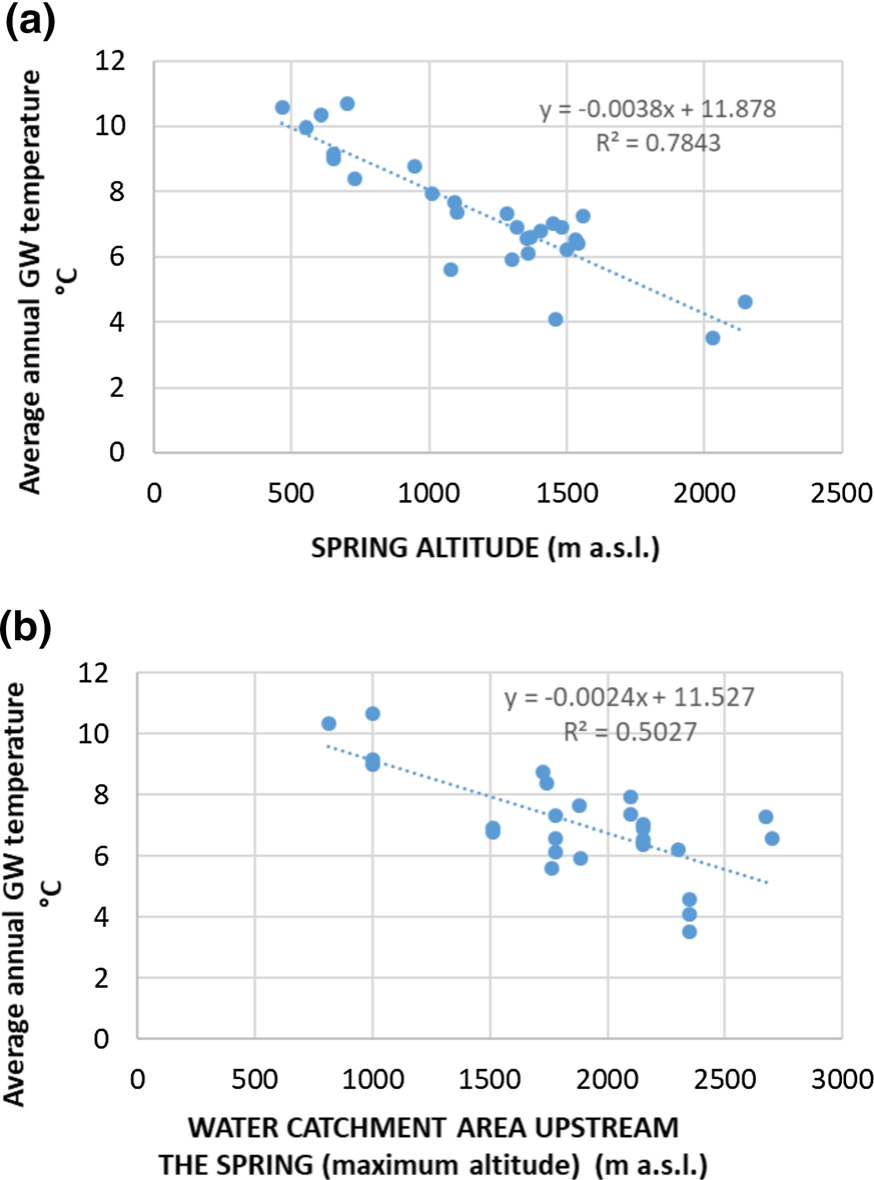
Correlation analyses between the average annual Tgw and the spring altitude (**a**) and the average annual Tgw and the maximum altitude of the WCS (**b**)

The biplot between the average annual Tgw and spring altitude (Fig. 4a) shows a good correlation (*R*^2^ = 0.78) between the parameters. More specifically, the Tgw of the spring decreases with the spring altitude. The biplot between the average annual Tgw in the spring and the maximum altitude of the WCS shows a weaker correlation (*R*^2^ = 0.50). The Tgw is generally higher with a WCS at low altitudes, and it decreases with increasing altitude (Fig. 4b).

#### Annual distribution of meteorological and spring parameters

**Fig. 5:**
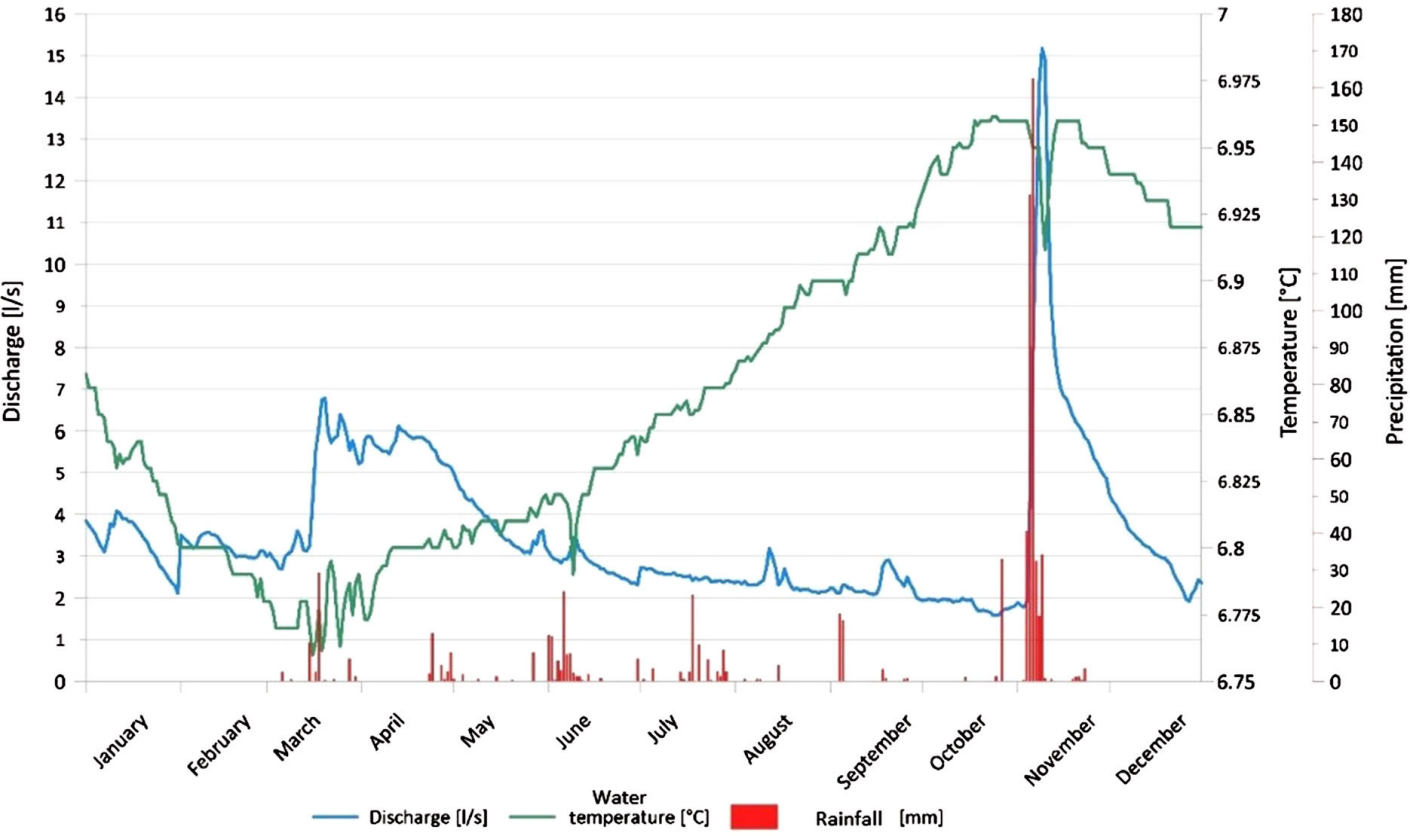
Distribution of daily Qgw, Tgw, and rainfall vs time in spring 20 (year 2011). In this spring, Qgw shows a rapid increase in correspondence with the main rainfall events

The visualization of springs and meteorological parameters in biplots (daily Qgw, Tgw, and rainfall vs time) showing the distribution of parameters during the year permitted us to observe very complex behaviours of the springs. Two spring behaviours are shown as examples in Fig. 5, 6 because they are representative of many springs.

**Fig. 6:**
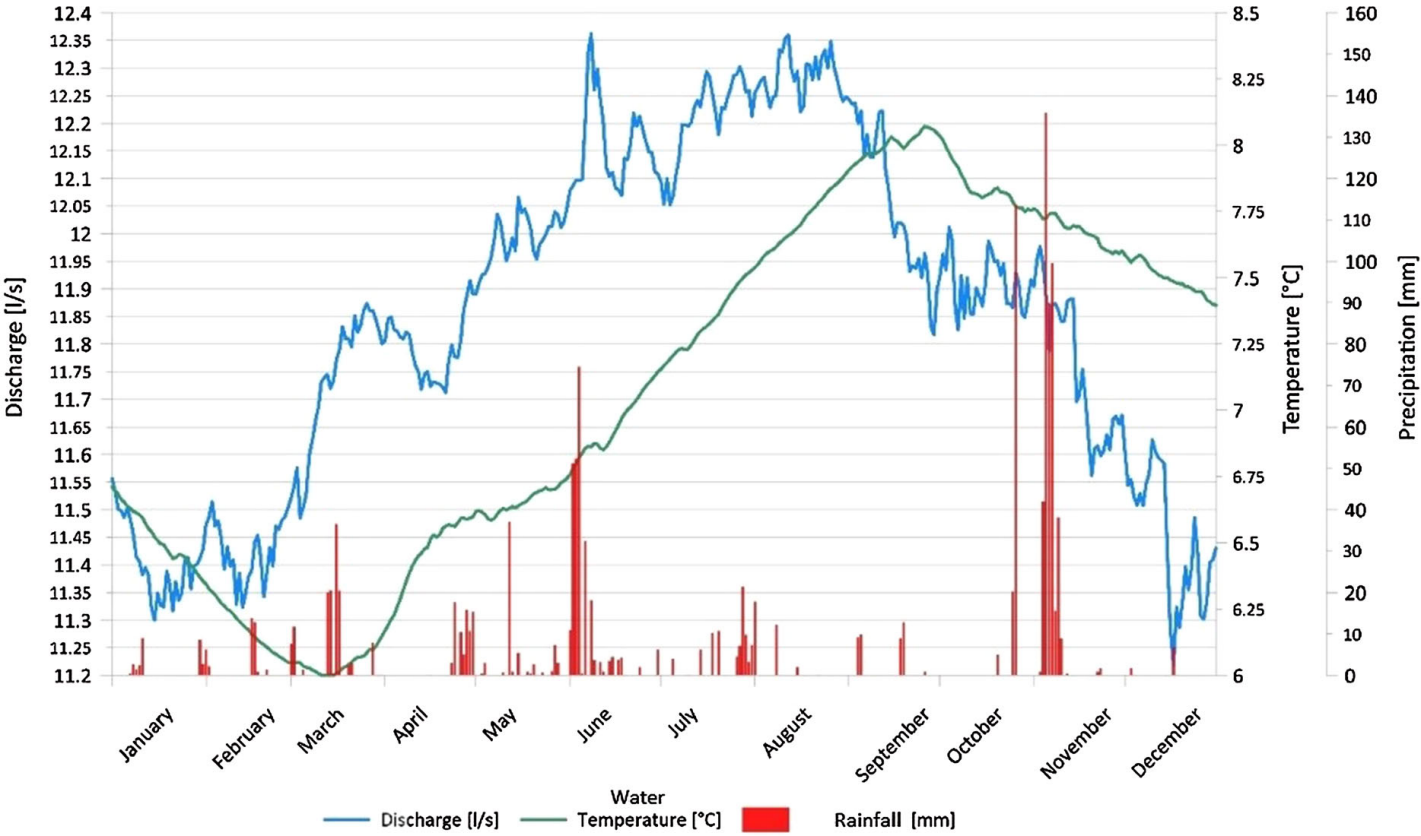
Distribution of daily Qgw, Tgw, and rainfall vs time in spring 15 (year 2011). In this spring, Qgw generally has a small increase in correspondence with the main rainfall events

Many analysed springs show a rapid increase in their Qgw in correspondence with the main rainfall events (e.g. Fig. 5). These springs generally have significant Qgw peaks in autumn (November) and spring (May–June), in correspondence to the main rainfall events of the year. Moreover, a Qgw peak in spring (starting in March) is usually present and is due to snow melting. The Tgw rises from spring through October–November; then, Tgw decreases, and it has a minimum at the end of winter. Aquifers associated with these springs have a very high permeability but a very small and limited saturated zone, which determines a rapid response to various outside inputs. In correspondence with the main rainfall events, springs respond quickly with an increase in their Qgw.

Other springs have their minimum Qgw in winter; then, the Qgw gradually increases with the beginning of springtime and reaches the maximum in the summer (e.g. Fig. 6). This behaviour is associated with snow melting in the recharge area. The Qgw generally has a small increase in correspondence with the main rainfall events. Sometimes the main Qgw peaks are delayed by weeks compared to the rainfall, depending on the features of the local hydrogeological context. Tgw generally has the same behaviour described in Fig. 5. These springs are associated with aquifers with a medium–good degree of permeability and a well-developed saturated area that allows the storage of groundwater.

#### Meinzer variability index

Most of the analysed springs (79%) are characterized by a high variability of Qgw (*R* > 100%). The remaining springs (21%) are subvariable springs, with a Meinzer variability index between 25 and 100% (Table 6).

**Table 6:** Meinzer variability indexes of the analysed springs. Spring numbers in accordance with Fig. 1. Qm—mean monthly discharge in the analysed period; R %—Meinzer variability index

Springs code	Qm	*R* %
1	0.74	250
2	35.59	39
3	1.84	239
4	5.63	166
5	4.2	225
6	8.76	412
7	10.03	346
8	9.91	173
9	30.52	232
10	1.61	201
11	20.58	83
12	13.55	147
13	16.68	98
14	9.39	91
15	12.32	67
16	20.76	38
17	0.48	129
18	0.58	378
19	1.78	172
20	3.49	247
21	13.44	288
22	9.47	354
23	6.74	714
24	6.56	175
25	8.66	222
26	1.48	874
27	6.97	211
28	4.11	329

**Fig. 7:**
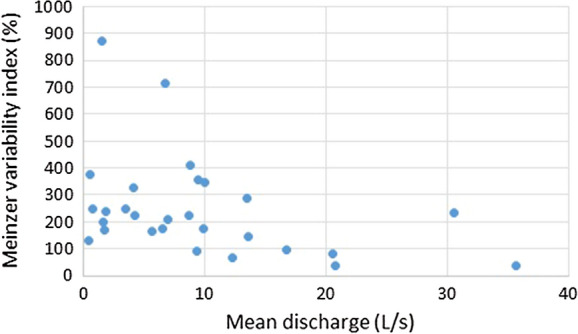
Mean monthly discharge vs the Meinzer variability index

The Qgw vs Meinzer variability index diagram shows that all the springs with low Qgw (less than 9 L/s) are variable springs (Fig. 7). Moreover, springs with the highest Qgw are variable or subvariable springs. None of the analysed springs can be classified as constant springs.

### Statistical analyses of trends

#### Tair trend

**Fig. 8:**
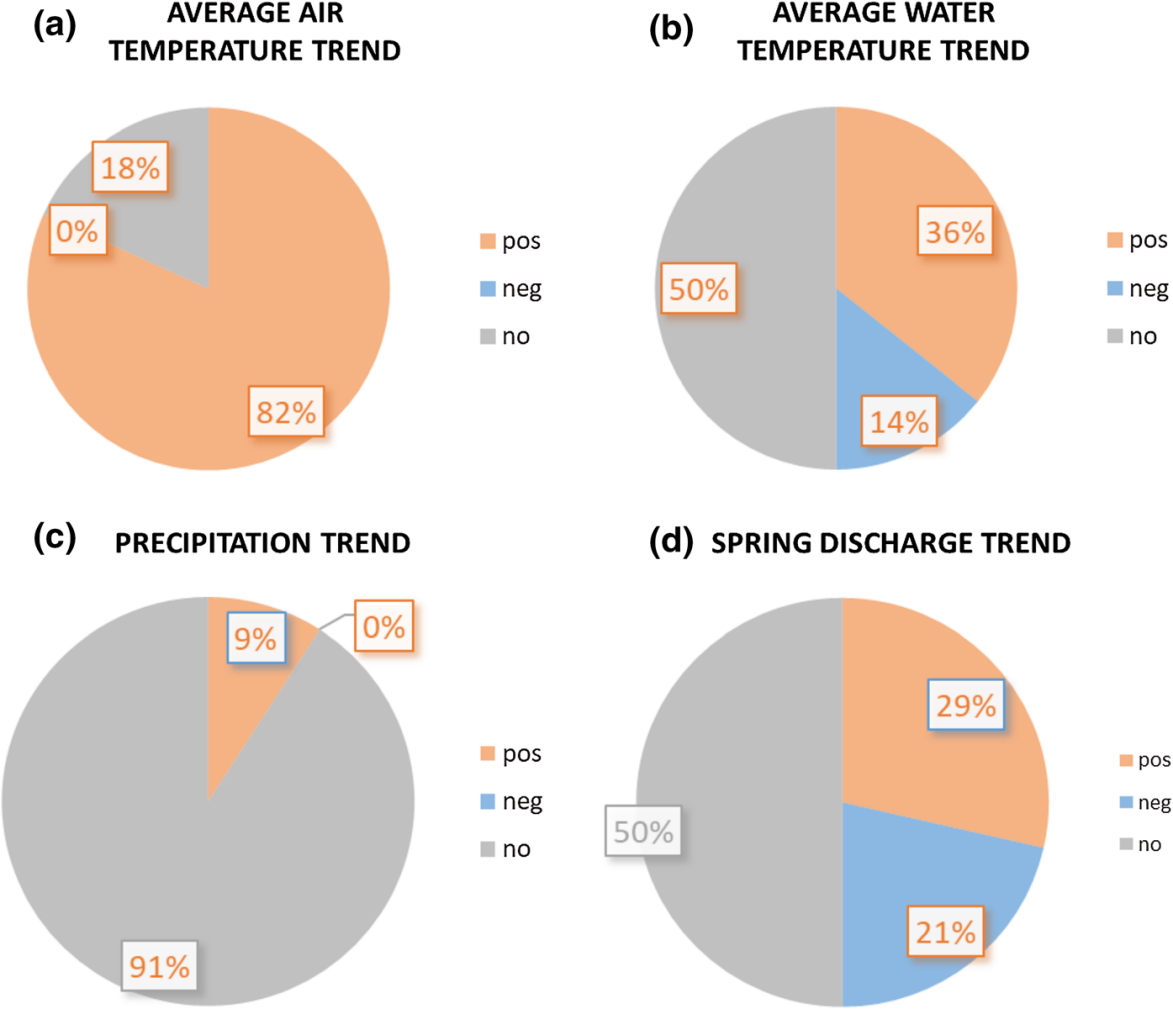
Synoptic image of the elaborated trends for average monthly Tair (**a**), average monthly Tgw (**b**), monthly cumulative rainfall (**c**) and average monthly Qgw (**d**). pos = positive trend; neg = negative trend; no = no trend

The average monthly Tair shows a positive trend almost everywhere (Fig. 8a). Indeed, the trend was observed in nine out of 11 weather stations, with the increase in Tair ranging from + 0.02 to + 0.03 °C/year. In contrast, no statistical trend of average Tair was observed at two weather stations (i.e. F and H), located in the towns of Barge and Vinadio, respectively (Cuneo Province) (Supplementary material 1). At the same stations, no trend was observed for the minimum and the maximum Tair. A positive trend for all the analysed parameters (i.e. average, maximum and minimum Tair) was calculated at only two weather stations (E and K), located in Turin and Cuneo Provinces, respectively. At the other stations, a positive trend was obtained for only one or two parameters. The average annual Tair versus time was also analysed using biplot and linear trend lines, and an overall increase between 0.35 and 1.79 °C was calculated in the monitored period.

#### Spring Tgw trend

A positive trend in the average monthly Tgw was observed in ten springs, with an increase in the average Tgw ranging from + 0.001 to + 0.02 °C/year (Supplementary material 2). A negative trend was evaluated for four springs, with a decrease ranging from −0.002 °C/year to −0.034 °C/year. Furthermore, 14 springs do not show a statistically significant trend. There is no particular spatial distribution of the observed trends.

The analysis of trends for the minimum and maximum Tgw trends also permitted us to notice a concordance of results in most of the investigated springs. More specifically, 13 springs show no trend for the minimum, average and maximum Tgw trend (i.e. springs 3, 6, 8, 11, 13, 14, 17, 18, 22, 24, 26, 27, and 28). The other four springs display a positive trend for the three evaluated parameters (i.e. springs 19, 20, 23, and 25). All of these springs are located in Cuneo Province. Finally, only one spring (spring 15) has a negative trend for all the analysed Tgw values.

#### Rainfall trend

The monthly cumulative rainfall shows no statistically significant trend in almost all the analysed weather stations (Fig. 8c). A trend, particularly a positive trend, is present at only one weather station (i.e. B), located in Varallo (Vercelli Province). The positive gradient is + 1.43 mm/year (Supplementary material 3).

#### Spring Qgw trends

The analysis of the trends for the average monthly Qgw highlights that half of the observed springs have no trend (i.e. springs 2, 8, 9, 11, 17, 18, 20, 21, 22, 23, 25, 26, 27, and 28) (Fig. 8d). However, despite the substantial stability of the rainfall amount, highlighted by the analysis of precipitation trends, a trend in the Qgw was observed in 14 out of 28 springs. More specifically, eight springs show a positive trend (i.e. 1, 4, 5, 12, 13, 16, 19, and 24), with a gradient ranging between + 0.003 l/s/year and 0.096 l/s/year. A negative trend was recognized in six springs (i.e. 3, 6, 7, 10, 14, and 15), with a gradient between −0.016 l/s/year and -0.24 l/s/year. It was not possible to identify a specific geographic distribution of the observed trends.

### Cross-correlation analysis

#### Cross-correlation between Tair and Tgw

**Fig. 9:**
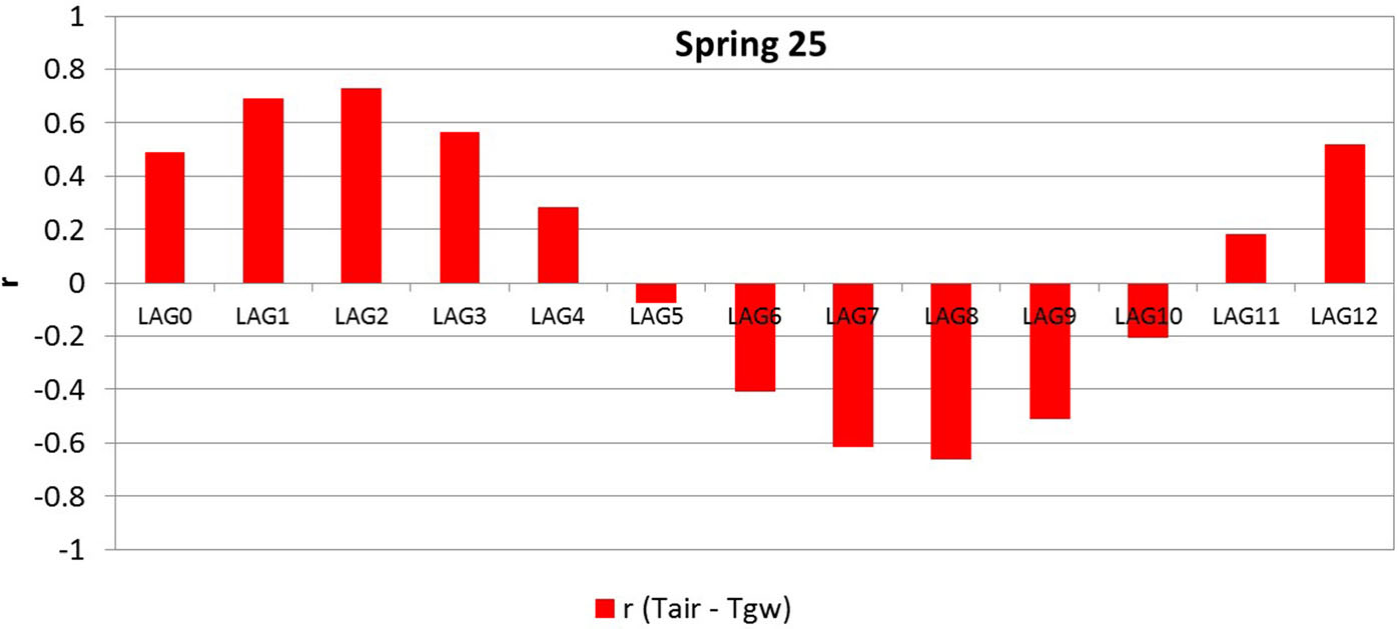
Example of a cross-correlogram of air temperature (Tair) and spring water temperature (Tgw) of spring 25. *r* = cross-correlation coefficient

The cross-correlation analysis between Tair and Tgw showed a medium–high positive correlation in the first months in 24 springs (r ranges between 0.45 and 0.98) and a low–very low correlation in four springs (r is between 0.05 and 0.20) (Table 7). The time lags generally vary between 0 and 3 months (only two show the best positive correlation in months 4 and 5). In Supplementary material 5, the coefficients of cross-correlation in the 28 analysed springs are presented. In Fig. 9, an example of a cross-correlogram between Tair and Tgw, relating to spring 25, is reported. It shows that the correlation coefficient r varies with annual cyclicity. In addition, the correlogram highlights a half-yearly cyclicity in which the correlation coefficient changes the sign: it assumes a maximum absolute value comparable to the maximal positives but with a negative sign.

**Table 7:** Cross-correlation coefficients computed between Tair and spring Tgw

Spring Code	LAG0	LAG1	LAG2	LAG3	LAG4	LAG5	LAG6	LAG7	LAG8	LAG9	LAG10	LAG11	LAG12
1	− 0.067	* − 0.08*	* − *0.056	* − *0.02	0.008	0.045	0.009	0.002	* − *0.005	* − *0.044	* − *0.03	* − *0.015	* − *0.049
2	− 0.009	* − 0.025*	* − *0.014	0.006	0.043	0.079	0.099	0.106	0.099	0.072	0.046	0.022	* − *0.007
3	0.981	0.83	0.485	0.023	* − *0.458	* − *0.808	* − *0.941	* − *0.825	* − *0.493	−0.02	0.456	0.802	0.944
4	− 0.005	0.089	0.163	0.207	0.193	0.118	0.037	* − *0.048	* − *0.118	* − *0.138	* − *0.125	* − *0.083	* − *0.001
5	0.357	0.659	0.797	0.724	0.451	0.056	* − *0.358	* − *0.659	* − *0.772	* − *0.686	* − *0.401	* − *0.026	0.344
6	0.613	0.693	0.572	0.313	* − *0.019	* − *0.36	* − *0.603	* − *0.686	* − *0.57	* − *0.305	0.048	0.39	0.638
7	0.599	0.713	0.607	0.338	* − *0.006	* − *0.349	* − *0.59	* − *0.674	* − *0.575	* − *0.341	* − *0.005	0.347	0.62
8	− 0.094	* − *0.113	* − *0.119	* − *0.019	0.053	0.051	0.18	0.133	0.093	0.056	* − *0.029	* − *0.15	* − *0.195
9	− 0.425	* − *0.295	* − *0.074	0.167	0.383	0.472	0.443	0.277	0.01	* − *0.205	* − *0.376	* − *0.446	* − *0.415
10	0.278	0.67	0.877	0.837	0.578	0.18	* − *0.274	* − *0.661	* − *0.847	* − *0.802	* − *0.553	* − *0.157	0.289
11	0.255	0.569	0.74	0.707	0.442	0.072	* − *0.283	* − *0.58	* − *0.747	* − *0.733	* − *0.538	* − *0.209	0.185
12	0.197	0.449	0.581	0.579	0.444	0.186	* − *0.061	* − *0.321	* − *0.465	* − *0.458	* − *0.376	* − *0.168	0.082
13	0.499	0.621	0.567	0.368	0.071	* − *0.234	* − *0.477	* − *0.596	* − *0.563	* − *0.382	* − *0.105	0.199	0.449
14	0.359	0.602	0.665	0.552	0.309	* − *0.025	* − *0.361	* − *0.584	* − *0.675	* − *0.582	* − *0.348	* − *0.03	0.31
15	0.479	0.619	0.582	0.385	0.089	* − *0.218	* − *0.463	* − *0.591	* − *0.55	* − *0.367	* − *0.111	0.184	0.45
16	− 0.038	* − *0.105	* − 0.152*	* − *0.139	* − *0.099	* − *0.072	0.038	0.1	0.117	0.176	0.133	0.044	* − *0.022
17	0.452	0.766	0.872	0.735	0.417	* − *0.008	* − *0.435	* − *0.745	* − *0.87	* − *0.758	* − *0.42	0.016	0.44
18	0.87	0.967	0.811	0.438	* − *0.045	* − *0.52	* − *0.849	* − *0.948	* − *0.803	* − *0.434	0.051	0.52	0.848
19	0.279	0.473	0.559	0.475	0.281	0.055	* − *0.219	* − *0.409	* − *0.522	* − *0.477	* − *0.298	* − *0.055	0.197
20	0.218	0.504	0.658	0.656	0.503	0.226	* − *0.116	* − *0.401	* − *0.541	* − *0.512	* − *0.339	* − *0.058	0.233
21	0.038	0.401	0.673	0.788	0.654	0.379	0.009	* − *0.354	* − *0.585	* − *0.668	* − *0.568	* − *0.322	0.039
22	− 0.141	0.162	0.412	0.544	0.506	0.355	0.125	* − *0.109	* − *0.313	* − *0.438	* − *0.452	* − *0.328	* − *0.076
23	− 0.315	* − *0.091	0.174	0.406	0.453	0.413	0.286	0.133	* − *0.046	* − *0.228	* − *0.369	* − *0.425	* − *0.276
24	0.445	0.714	0.795	0.649	0.329	* − *0.073	* − *0.466	* − *0.732	* − *0.798	* − *0.657	* − *0.343	0.045	0.435
25	0.486	0.693	0.728	0.563	0.279	* − *0.073	* − *0.405	* − *0.612	* − *0.66	* − *0.509	* − *0.205	0.181	0.516
26	0.605	0.85	0.863	0.634	0.259	* − *0.181	* − *0.566	* − *0.794	* − *0.826	* − *0.619	* − *0.246	0.202	0.593
27	0.787	0.879	0.719	0.359	-0.08	* − *0.519	* − *0.793	* − *0.856	* − *0.703	* − *0.347	0.081	0.483	0.761
28	0.096	0.478	0.722	0.789	0.638	0.315	* − *0.105	* − *0.484	* − *0.718	* − *0.757	* − *0.606	* − *0.301	0.088

#### Cross-correlation between spring Qgw and Tair

**Fig. 10:**
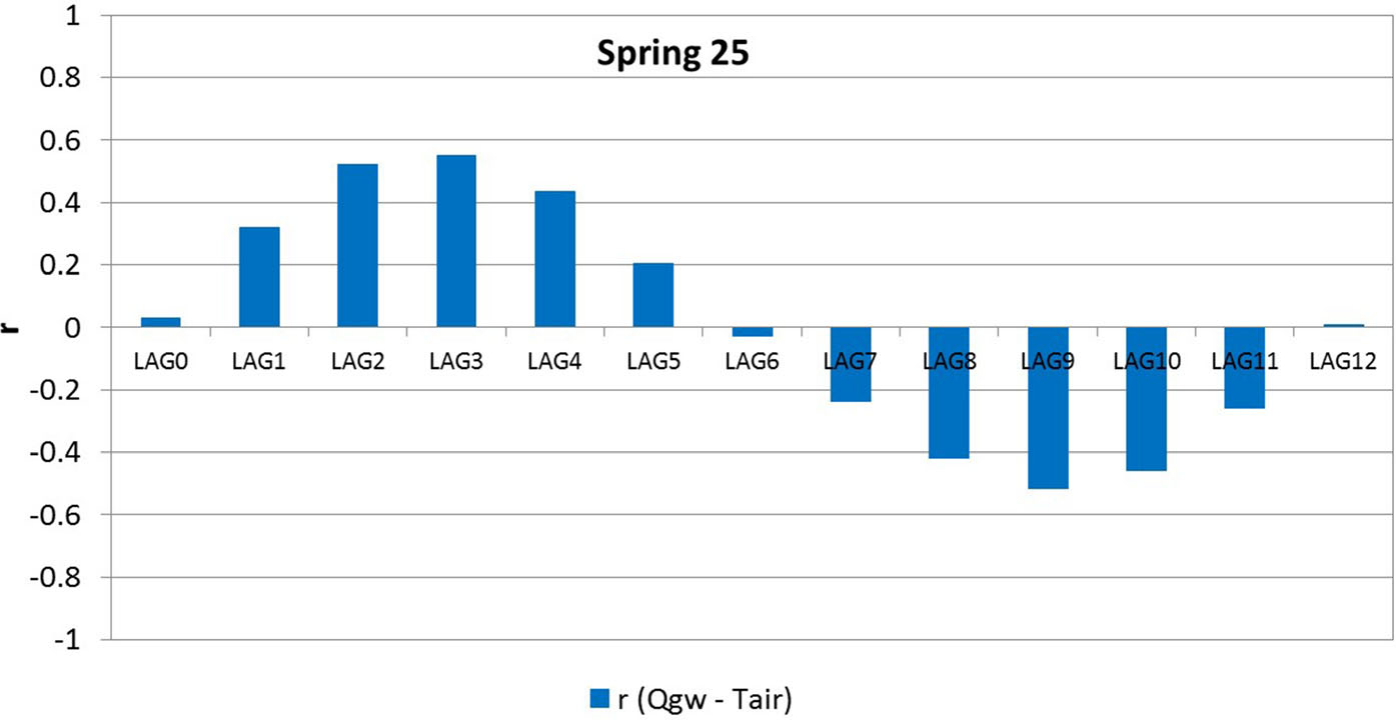
Example of the cross-correlogram of spring Qgw and Tair of spring 25. *r* = cross-correlation coefficient

Cross-correlation analysis between time series of monthly mean Qgw and Tair showed a positive best correlation coefficient at lags ranging between 1 and 3 months in 25 springs; the best correlation was observed at a lag of 0 months in two springs and at a lag of 4 months in only one spring (Table 8). In Supplementary material 5, the cross-correlation between Qgw and Tair in the 28 analysed springs is presented. An example of a cross-correlogram between Qgw and Tair, relating to spring 25, is reported in Fig. 10.

**Table 8:** Cross-correlation coefficients computed between Qgw and Tair

Spring Code	LAG0	LAG1	LAG2	LAG3	LAG4	LAG5	LAG6	LAG7	LAG8	LAG9	LAG10	LAG11	LAG12
1	0.006	0.047	0.069	0.088	0.09	0.073	0.058	0.025	− 0.017	− 0.038	− 0.044	− 0.037	− 0.011
2	0.245	0.274	0.233	0.126	− 0.018	− 0.157	− 0.234	− 0.268	− 0.222	− 0.12	0.001	0.133	0.23
3	− 0.038	0.045	0.088	0.109	0.087	0.06	0.011	− 0.068	− 0.106	− 0.114	− 0.115	− 0.084	− 0.024
4	0.383	0.459	0.397	0.205	− 0.074	− 0.296	− 0.379	− 0.386	− 0.299	− 0.151	0.051	0.249	0.4
5	0.488	0.449	0.306	0.074	− 0.18	− 0.403	− 0.513	− 0.446	− 0.25	0.023	0.276	0.445	0.491
6	0.16	0.279	0.318	0.284	0.126	− 0.074	− 0.255	− 0.368	− 0.386	− 0.299	− 0.147	0.013	0.19
7	0.205	0.295	0.294	0.229	0.063	− 0.136	− 0.306	− 0.398	− 0.384	− 0.259	− 0.083	0.084	0.249
8	0.232	0.288	0.269	0.186	0.049	− 0.136	− 0.226	− 0.243	− 0.188	− 0.092	− 0.019	0.088	0.168
9	0.35	0.372	0.293	0.158	− 0.051	− 0.27	− 0.385	− 0.374	− 0.23	− 0.038	0.149	0.302	0.363
10	0.321	0.385	0.341	0.203	− 0.001	− 0.23	− 0.362	− 0.371	− 0.287	− 0.141	0.005	0.184	0.307
11	0.468	0.515	0.449	0.217	− 0.085	− 0.349	− 0.491	− 0.471	− 0.358	− 0.204	0.009	0.231	0.384
12	0.389	0.488	0.467	0.273	0.042	− 0.202	− 0.368	− 0.394	− 0.392	− 0.296	− 0.133	0.061	0.244
13	0.151	0.247	0.258	0.212	0.099	− 0.053	− 0.171	− 0.21	− 0.196	− 0.164	− 0.109	− 0.013	0.089
14	0.051	0.081	0.086	0.055	− 0.013	− 0.09	− 0.134	− 0.148	− 0.131	− 0.099	− 0.065	− 0.028	0.004
15	0.211	0.245	0.216	0.09	− 0.088	− 0.23	− 0.277	− 0.261	− 0.145	0.034	0.138	0.181	0.198
16	0.137	0.169	0.157	0.106	0.038	− 0.04	− 0.097	− 0.121	− 0.115	− 0.072	− 0.001	0.077	0.14
17	0.285	0.339	0.31	0.168	− 0.023	− 0.227	− 0.347	− 0.364	− 0.289	− 0.16	− 0.027	0.135	0.255
18	0.1	0.162	0.167	0.124	0.061	− 0.033	− 0.106	− 0.162	− 0.194	− 0.153	− 0.077	0.022	0.116
19	0.092	0.317	0.478	0.508	0.386	0.151	− 0.106	− 0.314	− 0.446	− 0.45	− 0.35	− 0.131	0.11
20	− 0.131	0.11	0.283	0.363	0.331	0.204	0.085	− 0.035	− 0.176	− 0.319	− 0.39	− 0.313	− 0.128
21	− 0.026	0.216	0.375	0.384	0.281	0.107	− 0.015	− 0.127	− 0.207	− 0.306	− 0.324	− 0.224	− 0.021
22	− 0.065	0.174	0.362	0.394	0.315	0.138	0.004	− 0.115	− 0.216	− 0.325	− 0.389	− 0.276	− 0.075
23	− 0.018	0.208	0.35	0.375	0.283	0.13	− 0.024	− 0.151	− 0.25	− 0.351	− 0.326	− 0.22	0.004
24	− 0.112	− 0.054	− 0.04	− 0.01	0.048	0.042	0.094	0.113	0.135	0.079	− 0.034	− 0.081	− 0.091
25	0.032	0.319	0.52	0.55	0.434	0.203	− 0.026	− 0.238	− 0.418	− 0.515	− 0.457	− 0.258	0.008
26	− 0.151	− 0.002	0.126	0.174	0.174	0.103	0.08	0.041	− 0.033	− 0.114	− 0.205	− 0.22	− 0.146
27	− 0.003	0.219	0.372	0.384	0.27	0.087	− 0.067	− 0.177	− 0.278	− 0.324	− 0.327	− 0.23	− 0.005
28	0.033	0.327	0.522	0.575	0.444	0.209	− 0.04	− 0.278	− 0.455	− 0.542	− 0.479	− 0.275	0.019

## Discussion and conclusions

This study represents the first regional-scale investigation in the Piedmont alpine reliefs (NW Alps) of groundwater feature variation as a function of climate variability. More specifically, the analysis of 28 springs permitted us to highlight the existing correlations between spring discharge and some meteorological and topographic quantities in this mountainous area and to provide a preliminary framework of the impacts of climatic variability on the availability (discharge) and features (temperature) of the exploited water resources.

Trend analyses of springs and meteorological features helped us evaluate whether climatic variability had some consequences for water temperature and discharge in the studied period. The Tair shows a positive trend almost everywhere in the analysed period. In contrast, only ten springs show a positive trend for Tgw. The other springs generally show no trend (50% of the analysed springs) or a negative trend (14%). It was observed that the altitude of the springs has no influence on the typology of the trend (positive, negative or no trend). Moreover, the analysis of the Tair trend generally shows a higher positive gradient of Tgw (an average of 0.025 °C/year compared to that of Tgw, which was 0.007 °C/year). Because Tgw fluctuations of these springs are free from anthropogenic influences, the recorded variations can be attributed almost exclusively to climate variability.

As expected, the Tgw of the springs has a strong negative correlation with the spring altitude and the maximum altitude of the WCS; indeed, Tgw decreases with an increase in both of these parameters. These results are mostly due to the decrease in the average Tair with altitude. This is consistent with the fact that the temperature of groundwater is generally equal to the mean Tair above the land surface. However, some springs are located at a high altitude (higher than 2000 m), and snow melting at this altitude could affect groundwater very much, lowering its temperature. Consequently, the obtained correlation, quantified by the determination coefficient *R*^2^, is certainly inferior to the correlation in a hilly or plain area, located at low altitudes without snow melting.

Annual rainfall shows no statistically significant trend at almost all the analysed weather stations. Despite the substantial stability of the rainfall amount, only 50% of the analysed spring Qgw values do not show a statistically significant trend. The other springs show a positive (29% of the springs) or negative (21% of the springs) trend of discharge values. The decreasing Qgw may be due to the decrease in snow in the last 20 years (ARPA, 2013). Moreover, the decrease is larger than 15% of the average spring Qgw in only three springs. The Qgw rise could be a signal of more snow melting or the mobilization of water trapped in the permanently frozen layer. Consequently, it can indicate variation in the feeding of the natural mineral water springs connected to an increase in air temperature. Moreover, it was observed that the rate of Qgw, the specific geographic distribution, the altitude of the springs, and the surface of the WCS did not have a clear influence on the typology of the trend (positive, negative, or no trend).

Cross-correlation functions between time series (Tair, Tgw, and Qgw) permit us to prove the close relationship between the analysed parameters and to define the time lags (response time) between the time series. In particular, the correlation between Tair and Tgw generally shows a medium–high positive correlation in the first months, with a time lag between 0 and 3 months. This demonstrates a good relationship between Tair and Tgw. In particular, the cross-correlation coefficients are generally higher in the springs with the lowest maximum altitude of the WCS. This could indicate that Tgw is particularly influenced by Tair at low altitudes, while at high altitudes, Tgw is also affected by other factors, e.g. the temperature of water coming from snow melting and infiltrating towards the aquifer.

**Fig. 11:**
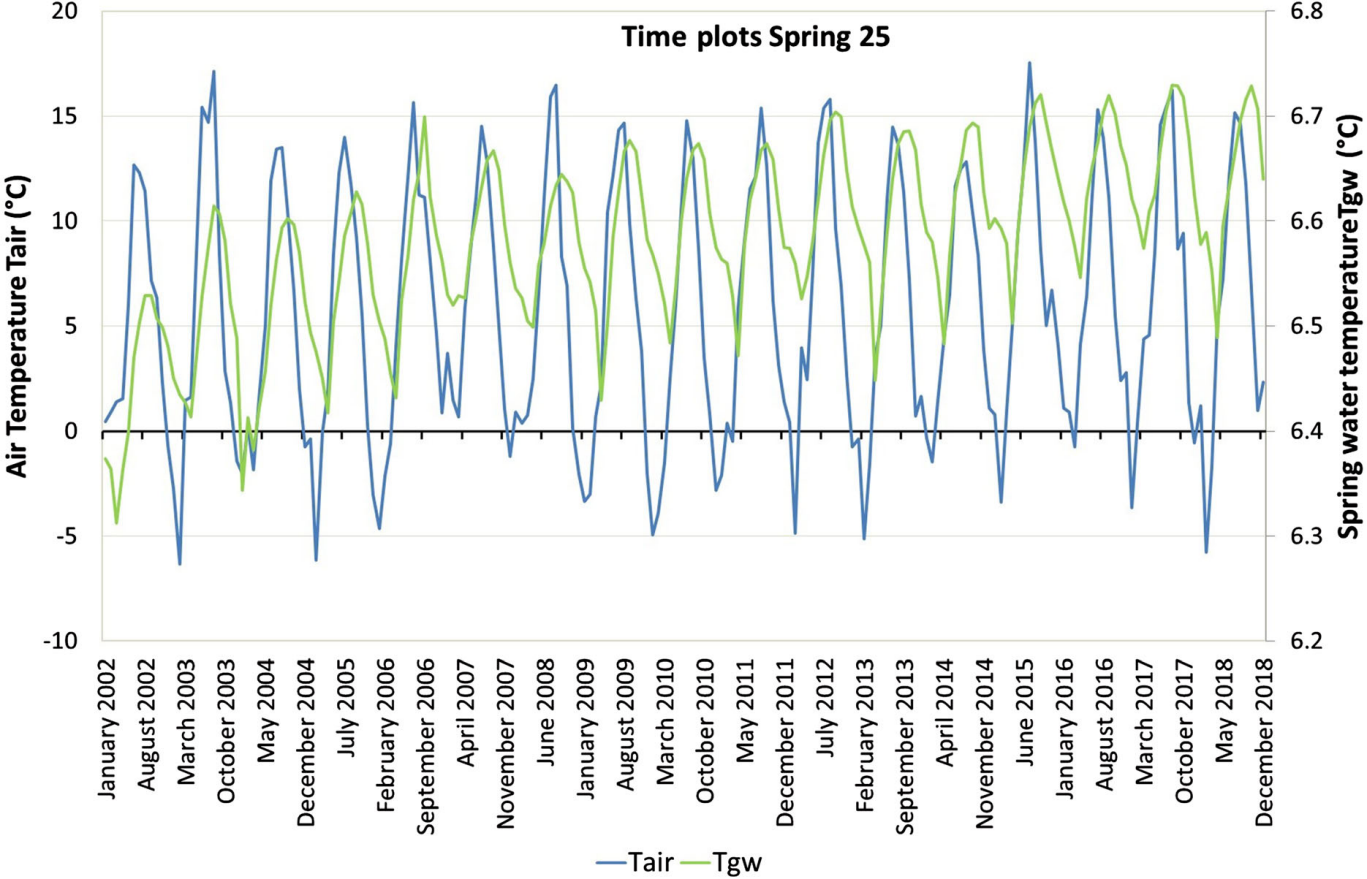
Time plots of spring water temperature (Tgw) and air temperature (Tair) of spring 25

To better observe the yearly and interannual behaviours of Tair and Tgw and their connections, these parameters were plotted vs time. Tair generally reaches the annual maximum in the months of July–August and a minimum in the winter months. This seasonality is detectable in all the thermometric measurement stations of the study area. Tgw, on the other hand, shows a similar cyclicity as Tair but shifted by a few months, depending on the analysed spring. Because this cyclicity is similar in many of the analysed springs, Fig. 11 reports the time plot of Tair and Tgw of spring 25 as an example of the distribution of these factors. In this spring, Tgw reaches a maximum in September–October, shifting by approximately 2 months compared to Tair.

**Fig. 12:**
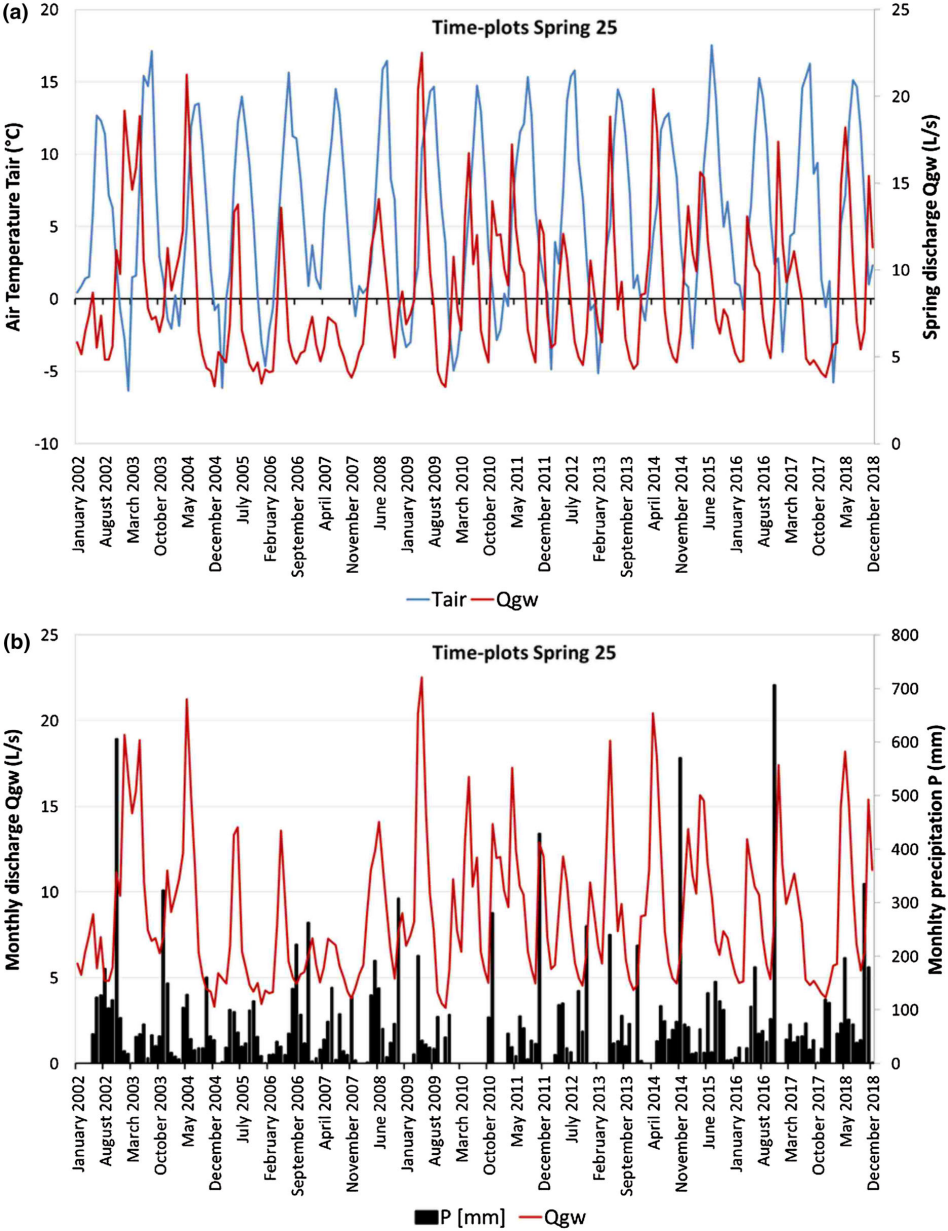
Time plot of monthly spring discharge (Qgw) and air temperature (Tair) (**a**) and time plot of monthly rainfall (P) and monthly spring discharge (Qgw) (**b**) of spring 25

On the other hand, the positive high correlation coefficient between Qgw and Tair in almost all the springs, with a lag between 1 and 3 months, highlights the strong relationship between these parameters. Figure 12 permits us to better observe their link, using spring 25 as an example. In particular, Tair is below 0 °C from December to March/April, and subsequently Tair starts to increase steadily until it reaches its maximum in July–August. The time plot of Qgw displays two peaks: a main peak generally in spring and a secondary peak generally in winter (Fig. 12a). Moreover, Qgw shows rapid growth starting in March, when the air temperature is generally above 0 °C, and it reaches its maximum in May. In this case, the rapid increase in Qgw is due not only to rainfall (see Fig. 12b) but probably also to snow melting in the recharge area. The secondary Qgw peak, in contrast, is clearly due to autumn rain, which assumes a very high value in November (Fig. 12b). The importance of snow melting is also supported by the altitude of the spring (1355 m a.s.l.) and the maximum altitude of the WCS (1780 m a.s.l.). Indeed, snow generally covers the study area at these altitudes during the winter (ARPA, 2013).

The understanding of the relationships between climate and spring discharge and temperature is increasingly important, especially in the context of global climate change. This study provides a framework of the possible impact of climatic variability on the availability and features of springs, representing the first step towards knowledge of the effects of climate change on mountain aquifers. In fact, time series are not long enough to analyse the performance of the springs in light of climate change, which, according to the IPCC (2014), consists of variations persisting for a longer period of time, typically decades or more.

Moreover, the study was applied to natural mineral water springs used for bottled water. Due to the increasing distribution and consumption of bottled natural mineral water in Italy, it is essential to have a framework for the current situation to comprise and recognize future developments and trends of groundwater quality and quantity.

Future insights will concern analyses of the longest time series of springs to clarify the long-term effects of global heating and groundwater recharge modification that could also affect water resource quality.

## Supplementary Information

Below is the link to the electronic supplementary material.

Supplementary file1 (DOCX 1814 KB)
