# Supplementary material for: Temperature and discharge variations in natural mineral water springs due to climate variability: a case study in the Piedmont Alps (NW Italy)
Source: Environ Geochem Health. 2021 Mar 3;44(7):1971–94. doi: 10.1007/s10653-021-00864-8 (PMC9177473; doi:10.1007/s10653-021-00864-8)
Supplement: Supplementary file 1 — Supplementary file1 (DOCX 1814 KB) [file 10653_2021_864_MOESM1_ESM.docx]

**Supplementary material**

Supplementary material 1

| Stations | Minimum Tair Trend | | | | Average Tair Trend | | | | Maximum Tair Trend | | | |
| --- | --- | --- | --- | --- | --- | --- | --- | --- | --- | --- | --- | --- |
|  | tau | p-value | Trend | Gradient [°C/year] | tau | p-value | Trend | Gradient [°C/year] | tau | p-value | Trend | Gradient [°C/year] |
| A | 0.043 | 0.353 | NO |  | **0.189** | **0.000** | **POS** | **+0.02** | **0.145** | **0.001** | **POS** | **+0.01** |
| B | **0.143** | **0.002** | **POS** | **+0.01** | **0.163** | **0.000** | **POS** | **+0.02** | 0.022 | 0.632 | NO |  |
| C | 0.031 | 0.501 | NO |  | **0.104** | **0.023** | **POS** | **+0.02** | **0.097** | **0.034** | **POS** | **+0.05** |
| D | 0.024 | 0.596 | NO |  | **0.125** | **0.006** | **POS** | **+0.03** | **0.085** | **0.048** | **POS** | **+0.04** |
| E | **0.165** | **0.000** | **POS** | **+0.01** | **0.236** | **0.000** | **POS** | **+0.03** | **0.094** | **0.041** | **POS** | **+0.05** |
| F | 0.025 | 0.588 | NO |  | 0.060 | 0.190 | NO |  | 0.081 | 0.236 | NO |  |
| G | **0.193** | **0.000** | **POS** | **+0.01** | **0.197** | **0.000** | **POS** | **+0.03** | 0.047 | 0.300 | NO |  |
| H | 0.048 | 0.482 | NO |  | 0.042 | 0.358 | NO |  | 0.096 | 0.161 | NO |  |
| I | 0.027 | 0.550 | NO |  | **0.159** | **0.001** | **POS** | **+0.03** | **0.114** | **0.013** | **POS** | **+0.06** |
| J | 0.041 | 0.375 | NO |  | **0.152** | **0.001** | **POS** | **+0.02** | 0.037 | 0.417 | NO |  |
| K | **0.131** | **0.004** | **POS** | **+0.02** | **0.124** | **0.007** | **POS** | **+0.03** | **0.160** | **0.000** | **POS** | **+0.06** |

Supplementary material 1: Trend analysis of minimum, average and maximum air temperature (Tair) at the considered weather stations of the Piedmont (NW Italy).

Supplementary material 2

| Springs | Minimum Tgw Trend | | | | Average Tgw Trend | | | | Maximum Tgw Trend | | | |
| --- | --- | --- | --- | --- | --- | --- | --- | --- | --- | --- | --- | --- |
|  | tau | p-value | Trend | Gradient [°C/year] | tau | p-value | Trend | Gradient [°C/year] | tau | p-value | Trend | Gradient [°C/year] |
| 1 | n.a. | n.a. | NO |  | **0.845** | **0.000** | **POS** | **+0.001** | n.a. | n.a. | NO |  |
| 2 | n.a. | n.a. | NO |  | **0.864** | **0.000** | **POS** | **+0.01** | n.a. | n.a. | NO |  |
| 3 | -0.036 | 0.445 | NO |  | 0.008 | 0.860 | NO |  | 0.064 | 0.173 | NO |  |
| 4 | **0.577** | **0.000** | **POS** | **+0.02** | **0.532** | **0.000** | **POS** | **+0.02** | **0.499** | **0.000** | **POS** | **+0.01** |
| 5 | **0.178** | **0.000** | **POS** | **+0.01** | **0.264** | **0.000** | **POS** | **+0.004** | **0.228** | **0.000** | **POS** | **+0.004** |
| 6 | 0.031 | 0.582 | NO |  | -0.063 | 0.290 | NO |  | -0.085 | 0.138 | NO |  |
| 7 | 0.004 | 0.948 | NO |  | 0.022 | 0.711 | NO |  | **-0.129** | **0.021** | **NEG** | **-0.02** |
| 8 | -0.085 | 0.161 | NO |  | -0.102 | 0.082 | NO |  | -0.075 | 0.216 | NO |  |
| 9 | **-0.127** | **0.025** | **NEG** | **-0.01** | **-0.194** | **0.000** | **NEG** | **-0.01** | -0.101 | 0.074 | NO |  |
| 10 | -0.102 | 0.060 | NO |  | **-0.115** | **0.030** | **NEG** | **-0.003** | -0.082 | 0.132 | NO |  |
| 11 | -0.181 | 0.070 | NO |  | -0.130 | 0.053 | NO |  | -0.106 | 0.115 | NO |  |
| 12 | **0.190** | **0.005** | **POS** | **+0.01** | **0.235** | **0.001** | **POS** | **+0.01** | **0.286** | **0.000** | **POS** | **+0.02** |
| 13 | 0.018 | 0.713 | NO |  | 0.065 | 0.178 | NO |  | 0.106 | 0.143 | NO |  |
| 14 | -0.077 | 0.143 | NO |  | -0.056 | 0.283 | NO |  | -0.002 | 0.965 | NO |  |
| 15 | **-0.236** | **0.000** | **NEG** | **-0.03** | **-0.163** | **0.001** | **NEG** | **-0.034** | **-0.112** | **0.029** | **NEG** | **-0.02** |
| 16 | n.a. | n.a. | NO |  | **-0.391** | **0.000** | **NEG** | **-0.005** | n.a. | n.a. | NO |  |
| 17 | 0.008 | 0.880 | NO |  | 0.029 | 0.581 | NO |  | 0.034 | 0.518 | NO |  |
| 18 | 0.060 | 0.191 | NO |  | 0.064 | 0.162 | NO |  | 0.051 | 0.264 | NO |  |
| 19 | **0.189** | **0.000** | **POS** | **+0.01** | **0.217** | **0.000** | **POS** | **+0.01** | **0.163** | **0.000** | **POS** | **+0.001** |
| 20 | **0.269** | **0.000** | **POS** | **+0.004** | **0.300** | **0.000** | **POS** | **+0.004** | **0.290** | **0.000** | **POS** | **+0.004** |
| 21 | 0.055 | 0.246 | NO |  | **0.100** | **0.033** | **POS** | **+0.004** | **0.083** | **0.042** | **POS** | **+0.002** |
| 22 | -0.020 | 0.673 | NO |  | -0.006 | 0.894 | NO |  | -0.041 | 0.380 | NO |  |
| 23 | **0.103** | **0.029** | **POS** | **+0.002** | **0.145** | **0.002** | **POS** | **+0.002** | **0.081** | **0.029** | **POS** | **+0.002** |
| 24 | -0.067 | 0.160 | NO |  | -0.044 | 0.355 | NO |  | -0.041 | 0.393 | NO |  |
| 25 | **0.373** | **0.000** | **POS** | **+0.01** | **0.438** | **0.000** | **POS** | **+0.006** | **0.440** | **0.000** | **POS** | **+0.006** |
| 26 | 0.054 | 0.251 | NO |  | 0.075 | 0.113 | NO |  | 0.068 | 0.149 | NO |  |
| 27 | 0.031 | 0.515 | NO |  | 0.014 | 0.768 | NO |  | 0.028 | 0.548 | NO |  |
| 28 | -0.071 | 0.138 | NO |  | -0.016 | 0.733 | NO |  | 0.020 | 0.684 | NO |  |

Supplementary material 2: Trend analysis of minimum, average and maximum water temperature (Tgw) in the 28 studied springs in the Piedmont Alps (NW Italy). n.a.: not analysed.

Supplementary material 3

| Stations | Rainfall Trend | | | |
| --- | --- | --- | --- | --- |
|  | tau | p-value | Trend | Gradient [mm/year] |
| A | 0.011 | 0.807 | NO |  |
| B | **0.133** | **0.039** | **POS** | **+1.43** |
| C | 0.032 | 0.485 | NO |  |
| D | 0.085 | 0.063 | NO |  |
| E | 0.061 | 0.182 | NO |  |
| F | 0.061 | 0.185 | NO |  |
| G | 0.074 | 0.105 | NO |  |
| H | 0.062 | 0.178 | NO |  |
| I | 0.077 | 0.093 | NO |  |
| J | -0.010 | 0.831 | NO |  |
| K | 0.009 | 0.838 | NO |  |

Supplementary material 3: Trend analysis of rainfall in the considered weather stations of the Piedmont (NW Italy).

Supplementary material 4

| Springs | Qgw Trend | | | |
| --- | --- | --- | --- | --- |
|  | tau | p-value | Trend | Gradient [l/s/year] |
| 1 | **0.232** | **0.000** | **POS** | **+0.006** |
| 2 | 0.015 | 0.745 | NO |  |
| 3 | **-0.158** | **0.001** | **NEG** | **-0.017** |
| 4 | **0.169** | **0.000** | **POS** | **+0.003** |
| 5 | **0.177** | **0.000** | **POS** | **+0.004** |
| 6 | **-0.236** | **0.000** | **NEG** | **-0.04** |
| 7 | **-0.226** | **0.000** | **NEG** | **-0.04** |
| 8 | 0.047 | 0.424 | NO |  |
| 9 | 0.073 | 0.189 | NO |  |
| 10 | **-0.192** | **0.000** | **NEG** | **-0.016** |
| 11 | 0.093 | 0.169 | NO |  |
| 12 | **0.205** | **0.003** | **POS** | **+0.038** |
| 13 | **0.285** | **0.000** | **POS** | **+0.096** |
| 14 | **-0.576** | **0.000** | **NEG** | **-0.24** |
| 15 | **-0.303** | **0.000** | **NEG** | **-0.034** |
| 16 | **0.480** | **0.000** | **POS** | **+0.08** |
| 17 | -0.027 | 0.601 | NO |  |
| 18 | -0.071 | 0.121 | NO |  |
| 19 | **0.238** | **0.000** | **POS** | **+0.004** |
| 20 | -0.008 | 0.867 | NO |  |
| 21 | -0.013 | 0.780 | NO |  |
| 22 | -0.071 | 0.132 | NO |  |
| 23 | 0.019 | 0.682 | NO |  |
| 24 | **0.227** | **0.000** | **POS** | **+0.04** |
| 25 | 0.054 | 0.250 | NO |  |
| 26 | 0.011 | 0.821 | NO |  |
| 27 | -0.079 | 0.093 | NO |  |
| 28 | 0.068 | 0.156 | NO |  |

Supplementary material 4: Trend analysis of the discharge (Qgw) in the 28 studied springs in the Piedmont Alps (NW Italy).

Supplementary material 5


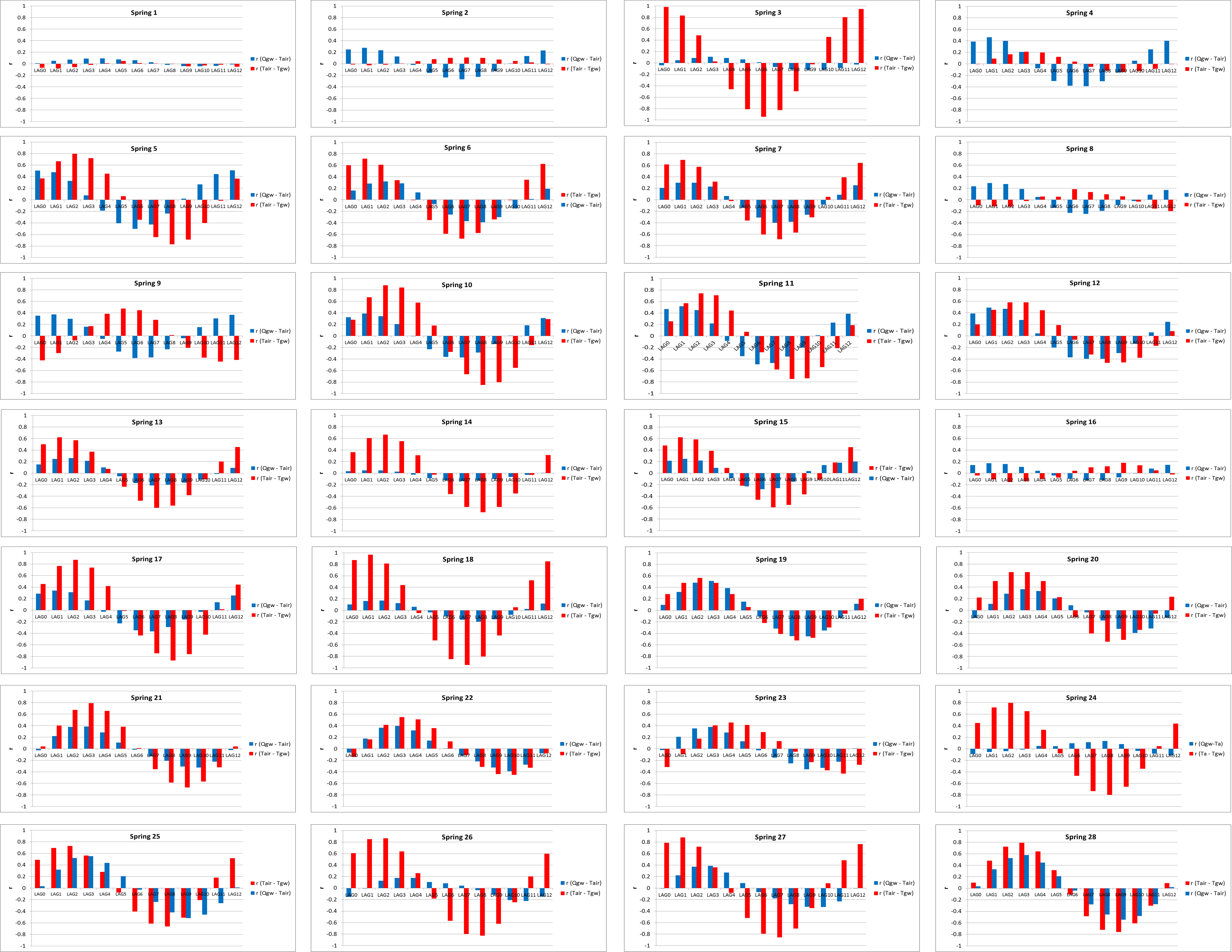


Supplementary material 5: Cross-correlograms of spring water discharge (Qgw) and air temperature (Tair) in blue and of air temperature (Tair) and spring groundwater temperature (Tgw) in red for the 28 analysed springs.
